# Critical Nutrients in Ketogenic Diets for Infants and Children Under Ten Years of Age—A Hypothetical Study

**DOI:** 10.3390/nu18101555

**Published:** 2026-05-14

**Authors:** Marc Assmann, Isabel Albrecht, Tobias Fischer

**Affiliations:** Center for Nutrition and Therapy (NuT), University of Applied Sciences Muenster, Corrensstraße 25, 48149 Muenster, Germany

**Keywords:** ketogenic diet, ketogenic metabolic therapy, nutritional therapy, epilepsy, infants, children, micronutrients, critical nutrients, optimized meal plans

## Abstract

Background: Ketogenic diets can treat drug-resistant epilepsy, even in early childhood. However, due to the severely restricted food selection, there is an assumed increased risk of inadequate micronutrient intake. Currently, the available data is limited. Methodology: Optimized daily meal plans were created for infants and children aged 1–9 years (physical activity level; PAL 1.6) in three ketogenic ratios (3:1, 2:1, 1:1). Compliance with reference values for micronutrients (≥95%) was analyzed using the reference values of the German and Austrian Nutrition Societies (DGE/ÖGE) and PRODI^®^ nutrition software (Germany). Results: Vitamin D never reached more than 25% of the reference values in any age group or ratio, and pantothenic acid consistently remained at around 40–70%. At the 3:1 and 2:1 ratios, the mean values for vitamins B_1_, B_2_, and B_12_, as well as for calcium, zinc, iron, and fiber, were all below 95% of the reference values. Although the 1:1 ketogenic ratio was more nutrient-dense, this only partially compensated for potential deficiencies. Conclusions: The results illustrate a limited micronutrient supply dependent on the ratio in ketogenic diets for infants and children. Careful food selection and nutritional therapy support are necessary to avoid potential nutrient gaps.

## 1. Introduction

A ketogenic diet (KD) is very high in fat and severely restricted in carbohydrates, mimicking the metabolic state of fasting and producing sustained ketosis. The resulting ketone bodies, primarily acetoacetate and 3-hydroxybutyrate (β-hydroxybutyrate; βHB), serve as an alternative energy source for the brain, heart, and skeletal muscles [1,2]. Introduced as early as the 1920s, KDs are one of the oldest non-pharmacological approaches in epilepsy therapy [3].

Today, KDs are considered an effective and established option for treating drug-resistant epilepsy in children and adolescents [1,4]. Several meta-analyses have approved the use of KDs as an independent treatment for reducing seizure frequency in infants, children and adolescents [5,6,7,8]. However, this is accompanied by a controversial debate concerning quality of life, growth, and development related to the diet [5]. According to German guidelines, they are also the preferred treatment for certain disorders of energy metabolism, including glucose transporter 1 (GLUT1) and pyruvate dehydrogenase complex (PDH) deficiencies [1]. There are various practical implementation options, including the classic KD (cKD), which usually has a fat-to-carbohydrate-and-protein ratio of 3:1 or 4:1 (fat: carbohydrates and protein), as well as more liberal forms, such as the modified Atkins diet (MAD), the low glycemic index treatment (LGIT), and the medium-chain triglyceride (MCT) diet [4,9,10].

KDs pose a particular challenge for children when they start eating solid foods. Due to their rapid growth and ongoing brain and skeletal development, children are susceptible to the consequences of insufficient micronutrient intake, especially when combined with poor diet adherence [9,11]. Studies on KDs in pediatric epilepsy patients have reported deficiencies in various micronutrients. Insufficient intake of vitamin D, calcium, folate, magnesium, iron, zinc, and selenium is particularly common and critical if no targeted supplementation is provided [9,12,13]. An evaluation of dietary records in children with epilepsy showed that a cKD does not meet reference values for many essential micronutrients. However, carefully planned, more liberal variants, such as the MAD, can achieve significantly higher coverage rates of national reference values [14]. However, corresponding analyses are limited, so statements about optimized, modern KDs are scarce.

National guidelines, international consensuses, and expert recommendations emphasize that KDs cannot be classified as nutritionally complete mixed diets and therefore require mandatory supplementation with vitamins, minerals, and trace elements [1,4,15,16]. At the same time, several studies describe possible effects on growth and bone metabolism. Prospective and retrospective studies report a slowdown in longitudinal growth with long-term ketogenic nutrition [9,17], underscoring the need for close nutritional monitoring [11]. Studies to date have mainly covered broad pediatric populations, such as those aged 1–16 or 0–18 years, without differentiating between specific age groups [9,13,14]. Additionally, the methodological focus of the available studies varies significantly. Some studies primarily analyze clinical parameters, such as growth or seizure reduction [12,13], while others consider nutrient calculations in relation to reference values [9,14]. Based on the limited available study data, Christodoulides et al. demonstrated both under- and overcoverage of individual micronutrients in clinical studies compared to national reference values. Depending on the type of diet and supplementation practice, elevated plasma levels of fat-soluble vitamins, especially vitamins A and E, were observed in a cKD and MCT-KD [12]. Liu et al. compared nutrient intake with Dietary Reference Intakes (DRIs) based on mandatory multivitamin supplements [18]. Therefore, it remains unclear to what extent modern, clinically applied KDs can achieve age-dependent reference values for micronutrients in different age groups without additional nutritional supplements.

Due to the limited data on potential micronutrient deficiencies, this study created optimized hypothetical ketogenic daily meal plans with different ratios for children aged 1 to 9 years. The plans were compared to the reference values of the German Nutrition Society (DGE) and the Austrian Nutrition Society (ÖGE) (DGE/ÖGE reference values). Based on these comparisons, potentially critical nutrients were identified, and practical conclusions were derived for nutritional therapy.

## 2. Materials and Methods

### 2.1. Data Acquisition

Optimized, hypothetical ketogenic daily meal plans for children aged 1–9 years old were developed (see exemplary ketogenic meal plans Table S1). Modeling was carried out for three age groups (1–3, 4–6, and 7–9 years) according to the DGE/ÖGE reference values and for the ketogenic ratios of 3:1, 2:1, and 1:1. Three daily meal plans were created for each age group and ratio, for a total of 27 plans included in the analysis.

The plans were created using PRODI^®^ Expert (version 7.3; Nutri-Science GmbH, Freiburg, Germany) based on food and nutritional data from the German Nutrition Database (BLS, version 3.02). The PRODI^®^ food search tool and the feature that enables comparisons between individual foods were used to identify keto-friendly, micronutrient-rich foods. Plans were created for each age group at a ratio of 3:1, which served as the basis for deriving the 2:1 and 1:1 ratios. Adjustments were made by modifying the amounts of fat, protein, and carbohydrates specifically. Energy supply was calculated using age-related DGE/ÖGE reference values: 1300 kcal for ages 1–3, 1600 kcal for ages 4–6, and 1900 kcal for ages 7–9. This calculation was based on an assumed PAL (physical activity level) value of 1.6, with a tolerance of ±5% of the respective reference energy. The amounts of fat were selected so that the ketogenic ratio would be within ±0.5 of the target value. For the 2:1 and 3:1 ratios, the maximum carbohydrate supply was set at 50 g/day, and for the 1:1 ratio, it was set at ≤60 g/day in accordance with an LGIT [4]. The age-dependent DGE/ÖGE reference values were used to calculate the daily protein amount for the 3:1 and 2:1 ratios (target range: ±5%). For the 1:1 ratio, higher amounts of protein (≤30 percent of daily energy; E%) were permitted in accordance with LGIT [19]. To ensure an even distribution of energy and nutrients throughout the day, the meal plans consisted of three main meals and one snack according to the German guideline for ketogenic nutritional therapies [1]. Water supply was based on age-dependent reference values [20].

After exporting the data records to Microsoft Excel (version 2510), the daily meal plans were reviewed to ensure they were complete. Missing or incomplete nutritional information was replaced with equivalent products from the BLS, and the data were adjusted accordingly to ensure consistency for all meal plans. For each meal plan, fiber and all available vitamins and minerals were calculated based on BLS data. Micronutrients without sufficient BLS data (chromium, selenium, and molybdenum) were excluded.

### 2.2. Statistical Analysis

Fiber and micronutrient coverage were reported as percentages of the respective reference values. A coverage level of 95% of the reference value was established as the threshold for an adequate supply, and lower values were considered as potentially critical. All calculations, as well as the determination of mean values, standard deviations, ranges, and percentage deviations from the reference values, were performed in Microsoft Excel.

## 3. Results

### 3.1. Energy and Macronutrients

The average energy supply across all ratios was 1275 ± 36 kcal for 1- to 3-year-olds, 1530 ± 8 kcal for 4- to 6-year-olds, and 1835 ± 44 kcal for 7- to 9-year-olds. This corresponds to approximately 96–98% of the reference values (target range: 95–105%).

Carbohydrate supply was below the specified upper limit of 50 or 60 g/day in all daily meal plans. The 3:1 ratio averaged 27.2 ± 4.7 g/day; the 2:1 ratio averaged 42.9 ± 7.3 g/day; and the 1:1 ratio averaged 58.5 ± 1.4 g/day. As the ratio became more stringent, the percentage of fat in the total energy supply increased, and the proportion of protein decreased. The average fat content was 88.9 ± 0.7 E% for the 3:1 ratio, 84.5 ± 1.0 E% for the 2:1 ratio, and 76.2 ± 1.0 E% for the 1:1 ratio. The protein amounts were 0.95 ± 0.07 g/kg body weight (bw) for the 3:1 ratio, 0.96 ± 0.07 g/kg bw for the 2:1 ratio, and 1.63 ± 0.04 g/kg bw for the 1:1 ratio. The calculated daily ketogenic ratios were 3.29 ± 0.20 (3:1), 2.32 ± 0.14 (2:1), and 1.41 ± 0.08 (1:1).

### 3.2. Nutrient Coverage Compared to Reference Values

The fiber content was below the reference values in all three ratios. There was an increase in fiber content as the ratio decreased: 3:1 (66 ± 20.2%, 47.9–94.1%); 2:1 (73.3 ± 20.8%, 50.5–118.6%); and 1:1 (83.2 ± 7.1%, 73.1–96.2%).

Vitamin D showed the lowest coverage of the reference values in all three ratios. The mean reference coverage was 20.0 ± 9.8% (7.8–36.9%) in the 3:1 ratio, 12.4 ± 9.9% (5.2–37.4%) in the 2:1 ratio, and 22.3 ± 9.1% (13.1–40.4%) in the 1:1 ratio. Among the B vitamins, vitamin B_1_ was notably below the reference values in the 3:1 and 2:1 ratios (3:1: 72.5 ± 12.5%, 50.9–91.9%; 2:1: 70.6 ± 11.3%, 52–92.1%), whereas the reference level was generally achieved in the 1:1 ratio (96.0 ± 19.2%, 69.8–119.6%). Similarly, vitamin B_2_ remained below 95% in the two restrictive ratios (3:1: 87.3 ± 16.0%, 69–120.2%; 2:1: 81.5 ± 15.8%, 68.1–119.1%), but notably higher values were observed in the 1:1 ratio (119.0 ± 24.0%, 90.7–162.5%). On average, pantothenic acid was below 95% in all ratios (3:1: 52.1 ± 17.3%, 32.8–87.5%; 2:1: 56.3 ± 12.8%, 34.7–72.8%; 1:1: 54.6 ± 19.9%, 33–95.3%). Vitamin B_12_ reached only about half of the reference intake in the 3:1 and 2:1 ratios (3:1: 54.5 ± 27.0%, 17.9–98.9%; 2:1: 55.5 ± 16.5%, 24.4–75.2%), while the 1:1 ratio provided an adequate supply on average (108.7 ± 38.3%, 46.7–173.2%).

In terms of minerals, the calcium level was slightly below the threshold at a 3:1 ratio (92.9 ± 7.3%; 83.8–104.6%), just above the threshold at a 2:1 ratio (98.5 ± 10.5%; 82.2–109.6%), and well above the threshold at a 1:1 ratio (109.5 ± 8.3%; 99.1–126.1%). Iron coverage was slightly insufficient in the 3:1 ratio (82.7 ± 16.2%, 61.2–111.9%), whereas the 2:1 and 1:1 ratios were above the reference values (2:1: 109.6 ± 19.4%, 74.8–140.7%; 1:1: 105.1 ± 28.7%, 85.6–179.2%). Zinc levels were consistently below the reference intake values in both the 3:1 (73.6 ± 15.8%, 50.1–94.6%) and 2:1 (83.4 ± 6.7%, 76.9–99.7%) ratios. However, in the 1:1 ratio, zinc levels reached values above 100% (117.3 ± 17.7%, 93.7–145.7%).

**Figure 1:**
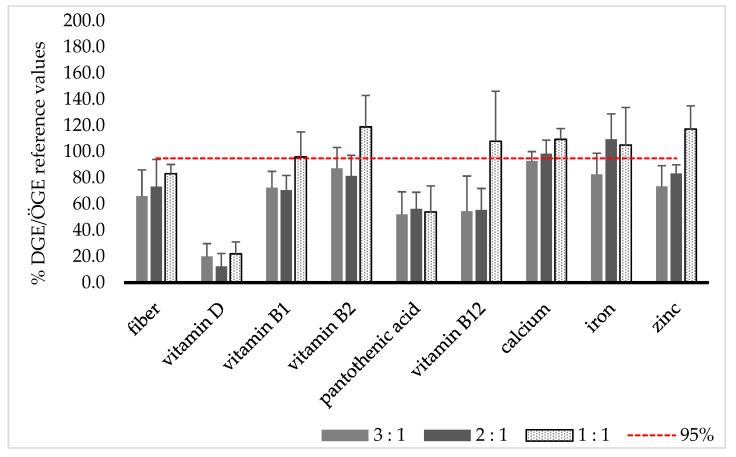
Percentage of the DGE/ÖGE reference values met for dietary fiber and analyzed micronutrients, differentiated by the ketogenic ratios 3:1, 2:1, and 1:1. The red line marks the 95% coverage level.

Overall, fiber, vitamin D, and pantothenic acid are insufficient in all ratios. Vitamin B_1_, vitamin B_2_, vitamin B_12_, and zinc are particularly lacking in the more restrictive ratios (3:1 and 2:1), as shown in Figure 1.

### 3.3. Nutrient Coverage by Age Group

The evaluation by age group revealed a slight tendency toward higher nutrient coverage with increasing age across all ratios (see Table 1). Eleven nutrients in the 3:1 ratio were below 95% of the reference values in the 1- to 3-year-old age group. This was still the case for nine nutrients in the 4- to 6-year-old age group and seven nutrients in the 7- to 9-year-old age group. In the 3:1 ratio, the most impacted vitamins were vitamin B_2_ (ages 1–3: 76.4 ± 11.5%; ages 4–6: 86.5 ± 11.0%; ages 7–9: 98.8 ± 19.9%), Vitamin B_3_ (ages 1–3: 85.6 ± 3.7%; ages 4–6: 95.0 ± 9.7%; ages 7–9: 111.5 ± 20.9%), pantothenic acid (ages 1–3: 38.7 ± 8.2%; ages 4–6: 50.2 ± 6.9%; ages 7–9: 67.4 ± 21.3%) and biotin (ages 1–3: 89.5 ± 7.9%; ages 4–6: 95.5 ± 13.9%; ages 7–9: 131.1 ± 36.8%) showed age-dependent increases in values. This tendency was particularly prevalent among the minerals for calcium (ages 1–3: 87.7 ± 5.1%; ages 4–6: 94.9 ± 3.9%; ages 7–9: 96.0 ± 10.5%) and for zinc (ages 1–3: 84.4 ± 14.7%; ages 4–6: 69.6 ± 17.9%; ages 7–9: 66.7 ± 13.6%).

At a 2:1 ratio, nine nutrients were below 95% of the reference values for the 1–3 and 4–6 age groups, while seven nutrients were below 95% of the reference values for the 7–9 age group. Among the vitamins, vitamin D (ages 1–3: 6.3 ± 1.6%; ages 4–6: 10.0 ± 1.3%; ages 7–9: 20.8 ± 14.7%), vitamin B_1_ (ages 1–3: 63.6 ± 10.1%; ages 4–6: 69.1 ± 9.8%; ages 7–9: 79.1 ± 11.3%), vitamin B_2_ (ages 1–3: 73.9 ± 9.8%; ages 4–6: 77.9 ± 8.3%; ages 7–9: 92.6 ± 23.4%), vitamin B_3_ (ages 1–3: 84.6 ± 8.1%; ages 4–6: 93.1 ± 11.5%; ages 7–9: 108.7 ± 15.5%), pantothenic acid (ages 1–3: 45.1 ± 10.7%; ages 4–6: 54.7 ± 9.3%; ages 7–9: 69.2 ± 3.2%) and biotin (ages 1–3: 91.6 ± 19.2%; ages 4–6: 94.2 ± 24.8%; ages 7–9: 109.4 ± 30.1%) showed increasing coverage of the reference values. Among minerals, zinc tended to decrease (ages 1–3: 87.8 ± 10.3%; ages 4–6: 82.1 ± 5.0%; ages 7–9: 80.4 ± 0.7%).

At a ratio of 1:1, the percentage of nutrients below 95% of the reference values was as follows: five in the 1–3 age group, three in the 4–6 age group, and four in the 7–9 age group. Vitamin B_12_ and pantothenic acid showed different trends: vitamin B_12_ increased with age (ages 1–3: 66.5 ± 20.7%; ages 4–6: 119.6 ± 16.9%; ages 7–9: 140.0 ± 28.8%), while pantothenic acid decreased (ages 1–3: 37.8 ± 4.2%; ages 4–6: 52.4 ± 11.2%; ages 7–9: 73.8 ± 21.6%). However, vitamin B_1_ decreased with age (ages 1–3: 102.3 ± 14.2%; ages 4–6: 99.1 ± 18.0%; ages 7–9: 86.6 ± 27.3%).

Overall, nutrient coverage tends to increase with age and decreased restrictiveness of the KD. This is particularly observable in the case of certain B vitamins and calcium. However, there are also isolated cases of declining values, such as with zinc.

## 4. Discussion

This analysis of hypothetical dietary meal plans indicates that a KD in infancy and childhood does not reliably provide adequate levels of several micronutrients. Vitamin D and pantothenic acid were particularly affected, with averages falling below the DGE/ÖGE reference values for all ratios and age groups. Additionally, critical values dependent on the ratio were found for fiber, vitamins B_1_, B_2_, and B_12_, calcium, iron, and zinc, with the number of potentially critical nutrients tending to increase as the ketogenic ratio increases. This decrease in micronutrient coverage as the KD becomes more restrictive is consistent with observations from other nutritional analyses of KDs [14,15,21].

In the dietary meal plans examined, fiber supply was below the DGE/ÖGE reference values for all age groups and ratios, with the 3:1 ratio showing the lowest coverage in particular. Interestingly, Volpe et al. reported that children with pharmaco-resistant epilepsy already exhibited significantly lower fiber intake compared to healthy peers and reference values prior to the initiation of a KD [22], indicating that reduced fiber intake may not be solely attributable to the dietary restrictions of KDs. However, current evaluations of the MAD and KD show that a targeted selection of fiber-rich, carbohydrate-reduced foods, such as vegetables, seeds, konjac-based products and ketogenic breads, can achieve a fiber intake within the reference values, at least with a MAD [14]. A reference value of over 80% was also achieved in the corresponding 1:1 ratio in the present analysis.

Vitamin D has repeatedly been identified as a critical nutrient in the context of a KD, given the observed suboptimal status of vitamin D in children on a KD [9,23,24]. This is consistent with the low potential supply observed in the present analysis. However, it should be noted that vitamin D deficiency and low nutritional intake are not problems that are exclusively associated with KD. National consumption data from Germany show that children, adolescents and adults already fall significantly below the recommended vitamin D intake values, even when consuming a normal mixed diet [25,26,27]. This is due to the limited availability of foods rich in vitamin D and the body’s insufficient synthesis through exposure to sunlight. International recommendations therefore emphasize the importance of supplementing vitamin D to ensure an adequate supply and prevent deficiencies, particularly rickets, in defined risk groups [28]. Therefore, the DGE/ÖGE reference values recommend vitamin D supplementation of 20 µg (800 IU) per day for all age groups over one year old, in cases of insufficient endogenous synthesis, for example, during the winter months [29]. However, vitamin D levels determined from dietary surveys and hypothetical dietary plans are not meaningful due to the low overall nutritional intake, and there is no correlation with corresponding blood values [30]. In contrast, supply levels of the other fat-soluble vitamins (A, E and K) were above the reference values in all ratios in the meal plans modeled here. This is consistent with literature on vitamins A and E, whereas only limited and inconsistent data on vitamin K are currently available [9,14,21].

A distinct ratio-dependent pattern emerged for B vitamins. Additionally, the more restrictive ratios revealed that biotin fell below the reference values in certain age groups, particularly younger children. This nutrient was not considered in other evaluations; therefore, a comparison with other data is not possible. Prudencio et al. and Velandia et al. in their evaluations of dietary records in children on a KD, reported insufficient intake of various B vitamins for the cKD. Meanwhile, liberal concepts such as MAD, particularly when using enriched formulas, significantly more often reached the reference values [13,21]. A detailed nutrient analysis by Tsang et al. showed that a carefully planned MAD can fully cover national reference values for most vitamins, including B vitamins. Deficiencies are more likely to occur in classic KDs with more stringent restrictions [14]. These results are consistent with the present analysis and demonstrate the impact of restrictiveness on B vitamin supply. Hypothetical daily meal plans by La Lenferna De Motte and Zinn also demonstrated that the nutrient coverage of KDs is closely related to the underlying food choices. Patterns predominantly consisting of unprocessed, protein- and nutrient-rich foods provide a better supply of B vitamins than patterns with a high proportion of specialized, commercially available, highly processed products that do not fall within the scope of food for special medical purposes (FSMP) [31]. The hypothetical meal plans presented here deliberately avoid using commercially available ketogenic substitute products to demonstrate to what extent the reference values can be achieved without them.

Calcium was deficient in the plans presented only in the 3:1 ratio. The comparatively better calcium supply in the 1:1 and 2:1 ratios is primarily due to the targeted inclusion of calcium-rich mineral water, which made a significant contribution to the total supply in these meal plans. In addition, the 2:1 ratio offered a slightly wider choice of foods due to its lower restrictiveness, which allowed additional calcium-rich foods to be integrated into the daily meal plans. Prospective data in children with pharmaco-resistant epilepsy showed a progressive decrease in bone mineral content under a KD, while studies on modified KDs in adults have described early changes in calcium and bone metabolism [24,32]. National and international consensus papers and guidelines therefore recommend specifically taking into account the age-dependent, nationally referenced values for calcium and supplementing calcium and vitamin D in an appropriate, low-carbohydrate form as part of a KD [1,4,16,33]. Other studies, such as that by Velandia et al., which was based on seven-day dietary records of children with pharmaco-resistant epilepsy, also found that calcium, iron and zinc intake were significantly below the reference values, particularly in the cKD, whereas a MAD with enriched formula achieved significantly better results [21]. Tsang et al. concluded from a model-based nutritional analysis of pediatric KD meal plans that calcium, iron and zinc intake without targeted supplementation regularly falls below international reference values [14]. Serum-based studies have shown that, without consistent supplementation, plasma zinc concentrations in can decrease during cKD or MCT-KD [12]. There is some discussion about using cast-iron pots to increase iron intake in KD. However, the benefits are unclear, particularly since an acidic environment in food promotes iron availability and the quantity of iron released from the pot is unknown [34,35]. The deficiencies described in the literature are consistent with the results of this analysis, particularly with regard to iron and zinc, which were potentially critical in most age groups and ratios. In contrast, this study showed more favorable overall reference value coverage for calcium, largely due to the targeted inclusion of calcium-rich mineral waters in the optimized daily meal plans, as described previously.

Overall, the results correspond to the expected pattern of decreasing nutrient density with increasing ketogenic ratio, as described for classic ketogenic 4:1 and 3:1 diets as well as for other severely carbohydrate-restricted diets [21,36,37]. More liberal variants allow for a significantly better approximation of reference values due to a wider variety of foods [21,31]. A high fat content, e.g., in the 3:1 ratio, reduces the proportion of vegetables, dairy products, eggs, and meat, i.e., food groups that provide the majority of B vitamins, minerals, and trace elements. This results in lower levels of vitamin B_1_, B_2_, B_12_, pantothenic acid, calcium, iron, and zinc [13,21,37]. However, as demonstrated with vitamin D, micronutrient deficiencies are not the only potential issue associated with KDs. A study of children and adolescents in Germany found that most participants had nutrient intakes, such as for calcium and iron, that were below the reference values. With the exception of folate, intake levels of water-soluble vitamins were adequate [25]. According to the Children’s Nutrition Survey to Record Food Consumption (KiESEL), pantothenic acid and iron intake were inadequate among toddlers (aged 1–2) and preschoolers (aged 3–5). Only among preschoolers was calcium intake also too low [27]. Internationally, infants and children are also known to consume lower than recommended amounts of iron and calcium [38,39]. However, it should be noted in this context that the data comes from nutritional surveys, meaning that actual dietary habits were recorded rather than optimized dietary patterns or recommendations. The results are based on optimized, hypothetical daily meal plans and therefore represent an ideal, controlled scenario. Practical experience shows that these plans are rarely implemented consistently in real-life settings, meaning lower micronutrient intakes are more frequently observed than in theoretically optimized meal plans, especially in the case of KD [21,36]. Additionally, the present analysis does not reflect individual food preferences, appetite, everyday eating situations, or disease- and age-related barriers, although guidelines explicitly recommend adapting to and considering preferences to ensure nutrient intake [4,15]. Furthermore, PRODI’s food search tools are not designed to be intuitive search functions. Instead, they are intended solely as a support tool, relying on the expertise of the nutritionist. Consequently, meal plan creation is influenced by unavoidable individual factors. Using the BLS leads to conservative estimates because fortified products and supplements were not considered. However, this is justified by the availability of data on 25 micronutrients in the BLS, for which national reference values are provided; these could not otherwise be fully included in the evaluation. Furthermore, the BLS is based on thorough analyses of the foods it contains. Despite its limitations, this analysis of hypothetical dietary meal plans provides a systematic assessment of nutrient coverage and can estimate intake within the framework of a modern KD.

## 5. Conclusions

The present analysis showed that even under optimized conditions, KDs do not consistently provide adequate amounts of certain micronutrients during the early years of childhood. Of particular note were the consistently insufficient supplies of vitamin D, pantothenic acid, and, depending on the ratio and age group, calcium, vitamins B_1_, B_2_, and B_12_, iron, zinc, and fiber. More restrictive ketogenic ratios had lower nutrient density than the more liberal 1:1 ratio. Therefore, a KD in childhood without targeted food selection and structured supplementation carries a potential risk of micronutrient deficiency. It is reasonable to consider mandatory supplementation, especially of vitamin D and calcium, as well as needs-based supplementation of other critical micronutrients and targeted incorporation of fiber into the diet. Careful menu planning that focuses on nutrient-dense foods, as well as accompanying nutritional therapy support, is crucial for identifying and addressing nutritional gaps related to ratios and age at an early stage. As part of nutritional therapy, a personalized micronutrient supplementation regimen should be determined that is tailored to the specific KD.

## Figures and Tables

**Table 1:** Mean and standard deviation (SD) of reference value coverage by age group and ratio (n = 3 plans per age group and ratio).

Nutrient	Ratio	Ages 1–3 (Mean in % ± SD)	Ages 4–6 (Mean in % ± SD)	Ages 7–9 (Mean in % ± SD)
fiber	3:1	71.0 ± 22.0	63.2 ± 19.9	63.7 ± 26.3
	2:1	73.8 ± 13.6	66.6 ± 13.7	79.6 ± 35.1
	1:1	82.3 ± 1.6	80.7 ± 5.4	86.5 ± 12.0
vitamin B_1_	3:1	75.1 ± 6.9	68.7 ± 17.0	73.9 ± 15.8
	2:1	63.6 ± 10.1	69.1 ± 9.8	79.1 ± 11.3
	1:1	102.3 ± 14.2	99.1 ± 18.0	86.6 ± 27.3
vitamin B_2_	3:1	76.4 ± 11.5	86.5 ± 11.0	98.8 ± 19.9
	2:1	73.9 ± 9.8	77.9 ± 8.3	92.6 ± 23.4
	1:1	99.5 ± 12.4	124.1 ± 24.5	133.3 ± 25.3
vitamin B_3_	3:1	85.6 ± 3.7	95.0 ± 9.7	111.5 ± 20.9
	2:1	84.6 ± 8.1	93.1 ± 11.5	108.7 ± 15.5
	1:1	112.5 ± 10.1	136.5 ± 7.1	151.7 ± 15.6
pantothenic acid	3:1	38.7 ± 8.2	50.2 ± 6.9	67.4 ± 21.3
	2:1	45.1 ± 10.7	54.7 ± 9.3	69.2 ± 3.2
	1:1	37.8 ± 4.2	52.4 ± 11.2	73.8 ± 21.6
biotin	3:1	89.5 ± 7.9	95.5 ± 13.9	131.1 ± 36.8
	2:1	91.6 ± 19.2	94.2 ± 24.8	109.4 ± 30.1
	1:1	107.1 ± 33.5	104.6 ± 34.7	145.2 ± 37.7
vitamin B_12_	3:1	58.6 ± 37.1	44.4 ± 23.1	60.5 ± 27.9
	2:1	56.6 ± 17.0	53.7 ± 5.3	56.2 ± 27.7
	1:1	66.5 ± 20.7	119.6 ± 16.9	140.0 ± 28.8
vitamin D	3:1	19.2 ± 9.8	16.0 ± 7.2	24.7 ± 13.3
	2:1	6.3 ± 1.6	10.0 ± 1.3	20.8 ± 14.7
	1:1	20.9 ± 7.0	20.7 ± 9.8	25.5 ± 12.9
calcium	3:1	87.7 ± 5.1	94.9 ± 3.9	96.0 ± 10.5
	2:1	98.8 ± 14.6	101.3 ± 7.3	95.3 ± 12.0
	1:1	105.6 ± 0.4	108.2 ± 9.8	114.7 ± 10.6
iron	3:1	70.5 ± 13.5	93.5 ± 16.0	84.1 ± 14.6
	2:1	104.0 ± 25.3	116.0 ± 23.8	108.7 ± 13.5
	1:1	92.4 ± 7.8	125.5 ± 46.6	97.4 ± 10.1
zinc	3:1	84.4 ± 14.7	69.6 ± 17.9	66.7 ± 13.6
	2:1	87.8 ± 10.3	82.1 ± 5.0	80.4 ± 0.7
	1:1	122.4 ± 14.1	118.0 ± 24.4	111.7 ± 19.2

## Data Availability

The data presented in this study are available on request from the corresponding author.

## Supplementary Materials

The following supporting information can be downloaded at: https://www.mdpi.com/article/10.3390/nu18101555/s1, Table S1: Examples of optimised, hypothetical ketogenic meal plans based on a 2:1 ketogenic ratio. Categorised by age groups (1–3 years, 4–6 years and 7–9 years) and by meal type (breakfast, snack, lunch and dinner).

## Abbreviations

The following abbreviations are used in this manuscript:

BLS	German Nutrition Database
bw	Body weight
cKD	Classic ketogenic diet
DGE	German Nutrition Society
DRIs	Dietary Reference Intakes
EsKiMo II	Nutrition study as a KiGGS module
FSMP	Food for special medical purposes
GLUT1-DS	Glucose transporter 1 deficiency syndrome
KiESEL	Children’s Nutrition Survey to Record Food Consumption
KiGGS	German Health Interview and Examination Survey for Children and Adolescents
KD	Ketogenic diet
LGIT	Low glycemic index therapy
MAD	Modified Atkins diet
MCT	Medium-chain triglyceride
ÖGE	Austrian Nutrition Society
PAL	Physical activity level
PDH	Pyruvate dehydrogenase
SD	Standard deviation
