# Supplementary material for: Critical Nutrients in Ketogenic Diets for Infants and Children Under Ten Years of Age—A Hypothetical Study
Source: Nutrients. 2026 May 14;18(10):1555. doi: 10.3390/nu18101555 (PMC13209885; doi:10.3390/nu18101555)
Supplement: Supplementary file 1 [file nutrients-18-01555-s001.zip › nutrients-4304990-supplementary.pdf]

Supplement 1

Table S1: Examples of optimised, hypothetical ketogenic meal plans based on a 2:1 ketogenic ratio. Categorised by age groups (1–3 years, 4–6 years and 7–9 years) and by meal type (breakfast, snack, lunch and dinner).

meal type	ages 1 – 3 years	ages 4 – 6 years	ages 7 – 9 years
**breakfast**	*Coconut-raspberry-yoghurt*<br>coconut milk, honey, coconut oil, coconut flakes, frozen raspberries, mascarpone, yoghurt, cocoa powder, mineral water	*Homemade bread roll with sausage and vegetables*<br>quark, mineral water, almond flour, rapeseed oil, sesame seeds, baking powder, cream, oat bran flakes, butter, salami, remoulade, tomatoes, cucumber, sparkling water	*Yoghurt with "muesli"*<br>walnuts, macadamia nuts, blueberries, mascarpone, yoghurt, soy drink, sparkling water
**snack**	*Sausage with vegetables*<br>Lyoner sausage, cucumber, mineral water	*Chocolate mousse*<br>whipped cream, sour cream, raspberries, dark chocolate, sparkling water	*Salami with vegetable sticks*<br>salami, peppers, cucumber, remoulade, sparkling water
**lunch**	*Stuffed peppers topped with cheese and bacon*<br>pork belly, mushrooms, rapeseed oil, iodised table salt with fluoride and folic acid, peppers, parsley, sour cream, Gouda, tomato purée, mineral water	*Ratatouille*<br>carrots, peppers, courgettes, rapeseed oil, peeled tomatoes, minced pork/beef, sour cream, onions, iodised table salt with fluoride and folic acid, pepper, sparkling water	*Cauliflower and carrot patties*<br>egg, mixed herbs, iodised table salt with fluoride and folic acid, rapeseed oil, almond flour, onions, cauliflower, carrots, hollandaise sauce, sparkling water
**dinner**	*Courgette-mozzarella-patties*<br>crispbread, mozzarella, basil, olive oil, rapeseed oil, iodised table salt with fluoride and folic acid, margarine, courgette, mayonnaise, tomatoes, mineral water	*Broccoli soup*<br>broccoli, crispbread, sour cream, vegetable stock, pepper, iodised table salt with fluoride and folic acid, mozzarella, onions, sunflower seeds, rapeseed oil, mixed herbs, spinach leaves, sparkling water	*Vegetable casserole*<br>chickpeas, iodised table salt with fluoride and folic acid, spinach, peas, feta cheese, boiled potatoes, mixed herbs, sour cream, onions, rapeseed oil<br><br>*Avocado and chocolate smoothie*<br>avocado, hazelnut oil, cow's milk, cocoa powder, mineral water, hazelnut butter, sparkling water
